# Evaluation of the Simultaneous Production of Xylitol and Ethanol from Sisal Fiber

**DOI:** 10.3390/biom8010002

**Published:** 2018-01-10

**Authors:** Franklin Damião Xavier, Gustavo Santos Bezerra, Sharline Florentino Melo Santos, Líbia Sousa Conrado Oliveira, Flávio Luiz Honorato Silva, Aleir Joice Oliveira Silva, Marta Maria Conceição

**Affiliations:** 1 Departamento de Química, PPGQ/CCEN, Universidade Federal da Paraíba, João Pessoa 58051-970, Brazil; franklind19@gmail.com (F.D.X.); gustavo_bezerra31@hotmail.com (G.S.B.); aleirsilva1@gmail.com (A.J.O.S.); 2 Departamento de Engenharia Química/CT, Universidade Federal da Paraíba, João Pessoa 58051-900, Brazil; sharlinefm@hotmail.com (S.F.M.S.); flavioluizh@yahoo.com.br (F.L.H.S.); 3 Unidade Acadêmica de Engenharia Química/CCT, Universidade Federal de Campina Grande, Campina Grande 58429-140, Brazil; libiaconrado@yahoo.com.br; 4 Centro de Tecnologia e Desenvolvimento Regional (CTDR)/Departamento de Tecnologia de Alimentos (DTA)/IDEP, Universidade Federal da Paraíba, Av. dos Escoteiros, sn. Mangabeira VII, João Pessoa 58058-600, Brazil

**Keywords:** polysaccharides, hemicellulose, bioproducts, xylose

## Abstract

Recent years have seen an increase in the use of lignocellulosic materials in the development of bioproducts. Because sisal fiber is a low cost raw material and is readily available, this work aimed to evaluate its hemicellulose fraction for the simultaneous production of xylitol and ethanol. The sisal fiber presented a higher hemicellulose content than other frequently-employed biomasses, such as sugarcane bagasse. A pretreatment with dilute acid and low temperatures was conducted in order to obtain the hemicellulose fraction. The highest xylose contents (0.132 g·g^−1^ of sisal fiber) were obtained at 120 °C with 2.5% (*v*/*v*) of sulfuric acid. The yeast *Candida tropicalis* CCT 1516 was used in the fermentation. In the sisal fiber hemicellulose hydrolysate, the maximum production of xylitol (0.32 g·g^−1^) and of ethanol (0.27 g·g^−1^) was achieved in 60 h. Thus, sisal fiber presents as a potential biomass for the production of ethanol and xylitol, creating value with the use of hemicellulosic liquor without detoxification and without the additional steps of alkaline pretreatment.

## 1. Introduction

Recent years have seen a search for new energy sources, both to replace fossil fuels and to reduce the environmental impacts of their use. In this search, lignocellulosic materials have appeared to be an alternative in producing biofuels and high value-added derivatives. This biomass is considered to be the largest source of renewable carbon on the planet, and it stands out due to its low cost, non-food application and the continuous source of biodegradable structural materials [1].

The lignocellulosic materials consist primarily of cellulose, hemicellulose, and lignin [2]. Cellulose can be used in different types of industries, such as the paper, pharmaceuticals, and textile industries. From the hemicellulose present in these materials, it is possible to obtain a hydrolysate rich in fermentable sugars, such as xylose, from which bioproducts such as ethanol and xylitol can be synthesized [3,4,5].

Sisal (*Agave sisalana*) is a plant widely cultivated in Brazil. This can be attributed to the wide application of its fibers and to its persistence in dry climates. Sisal fibers have good mechanical properties, high hardness, resistance to physical wear, and are biodegradable and renewable; therefore, the industrial application of sisal has been growing in various sectors, such as in the chemical industry. In Brazil, sisal plantations are concentrated in the states of Paraiba and Bahia, with a planted area of 154,000 ha and producing 800 kg/ha of sisal fiber [6,7]. According to the National Supply Company [8], Brazil is the world’s largest producer and exporter of sisal fiber, accounting for 50% of the world’s total production. This is equivalent to 91,100 tons and worth approximately $123.9 million. The cost of the biomass is low; it is not used as a food source; and about 90% of its composition is fermentable sugars such as xylose and glucose, which is 10% more than corn stover and sugarcane bagasse [7,9]. Thus, sisal is a material that has the potential to serve as a feedstock for the production of xylitol and ethanol.

When using biomass as feedstock, for example, in biorefineries, it is important to utilize all of its components for added value. The hemicellulosic fraction is an important part of the lignocellulosic materials; it is composed of pentoses (xylose and arabinose), hexoses (glucose, mannose, and galactose), and a small amount of uronic acids, mainly as side groups. Xylose is the primary carbohydrate component of hemicellulose present in sisal and can be converted to xylitol and ethanol through a fermentation process [10,11]. Furthermore, obtaining a bioproduct from an intermediate pretreatment step (alkaline or acid) prevents the need for a subsequent step, which would involve either acidic or enzymatic hydrolysis. In this sense, the simultaneous production of xylitol and ethanol from the hemicellulose fraction of sisal fiber is especially important, as it allows two products of great industrial interest to be obtained using a reduced number of steps, fewer reagents, and less energy [10,12,13,14].

In order to obtain bioproducts from lignocellulosic biomass, chemical and physical-chemical methods are used to selectively recover components such as hemicellulose and lignin, facilitating the solubility of sugars. The percentage of lignin in the sisal varies from 2 to 20%, which is less than other materials such as bamboo, sugarcane bagasse, and coconut [11]. Acid pretreatment has been widely used for the recovery of components, due to acid primarily affecting the polysaccharides that constitute hemicelluloses. A disadvantage of this method is reactor corrosion and monosaccharide degradation. The degradations of xylose and glucose produce inhibitors of the microbial fermentation furfural and 5-hydroxymethylfurfural (HMF), respectively. In addition to these, the production of other inhibitors also occurs, such as phenolic compounds, acetic, and formic and levulinic acids. The formation of these compounds is linked to factors such as high acid concentrations and high temperatures [11,15,16]. A solution to avoiding such problems is the use of diluted acid in concentrations lower than 10% (*v*/*v*) and the use of temperatures lower than 140 °C, as these generate smaller amounts of fermentative inhibitors. In addition, it produces a good amount of monomeric sugars from the hemicellulosic fraction, whose solubilizations need to be optimized [17].

Xylitol is among the top 12 value-added chemicals considered by the Department of Energy/USA that can be produced from plant biomass [9]. Xylitol is a polyalcohol, formula C_5_H_12_O_5_, which has a hydroxyl attached to each carbon atom, and high sweetening power and solubility [17,18]. Compared to sucrose, xylitol has almost the same sweetening power, but it also presents the advantage of lower caloric content (about 40% fewer calories than the amount found in sucrose) [19]. Due to being a less caloric sweetener, xylitol is used as a substitute for sugar in the food industry and also in the diets of patients with insulin deficiency, due to it being metabolized differently [20,21,22]. In addition, xylitol is largely utilized in the dental industry, as a result of its anti-cariogenic and biomedical properties, making it an interesting ingredient in the formulation of functional foods [23].

Industrially, xylitol is synthesized from the chemical reduction of xylose through hydrogenation with a nickel catalyst. This method demands high temperature and pressure conditions, and is characterized by high production costs and low yields. Xylitol can also be obtained through biotechnological procedures in which the xylose present in a biomass is converted into xylitol using specific microorganisms, especially *Candida* spp., which have a high yield of xylitol under conditions of limited oxygen [24].

Second-generation ethanol produced from lignocellulosic biomass is considered to be the biofuel with the greatest potential to replace petroleum-based fuels [25]. However, the costs associated with this technique are still high, which is a disadvantage. Nevertheless, if it could be simplified by removing a few steps, the process might become advantageous, for reasons such as the reduced need for alkaline pre-treatment and the production of biofuels from the hemicellulosic fraction obtained through acid pretreatment.

This work aimed to evaluate the hydrolyzed liquor of the hemicellulose fraction of sisal fiber as substrate in the simultaneous production of xylitol and ethanol, analyzing the best pretreatment conditions and the changes in the lignocellulosic matrix.

## 2. Results and Discussion

### 2.1. Acid Pretreatment

The acid pretreatment was conducted in order to obtain the hemicellulose fraction of the sisal. The study aimed to determine the concentration of dilute sulfuric acid and the temperature which were most efficient in extracting the xylose (Table 1).

**Figure 1:**
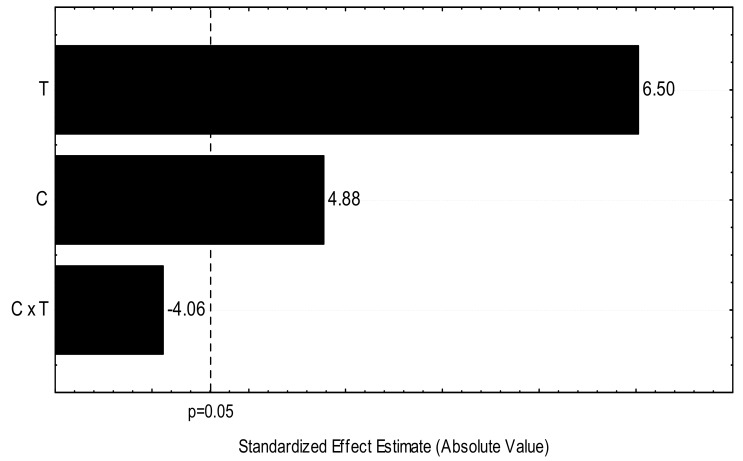
Pareto graph of the experimental design shows the parameters T (temperature), C (acid concentration), and CxT (temperature and acid concentration interaction) in relation to the xylose response variable, at the 95% confidence level.

The statistical analysis [26] indicated that the condition of higher-acid concentration and higher temperature was more suitable for xylose extraction, as shown in the Pareto charts in Figure 1.

The Pareto graph is widely used to represent the significance of parameters and their interactions in experimental design. In Figure 1, the horizontal bars show the importance of the factors analyzed in descending order, so that any parameter that exceeds the dashed line is significant (temperature, acid concentration) for the xylose response variable. The interaction TC (temperature × acid concentration) was not significant. The model was statistically significant at a confidence level of 95%, and it was confirmed by the *F*-test (Table 2).

According to the equation presented in Table 2, it is possible to say that the first-order model was statistically significant for xylose removal, because it presented an *F*-calculated/*F*-tabulated ratio higher than 1. Through the applied linear regression model, it was possible to determine with 95% confidence that the interaction between the two variables studied was not significant, and that the first-order model describes the efficacy of pretreatment in the xylose removal, with the parameters studied (temperature and acid concentration) being statistically significant.

The response surfaces for the sisal fiber indicated that the highest xylose content (0.132 g·g^−1^ of sisal fiber) was obtained at the maximum levels, which corresponded to the temperature of 120 °C and 2.5% (*v*/*v*) of sulfuric acid. The amounts of HMF and furfural were below toxicity levels. Though the focus of the treatment was the solubilization of the sugars present in the hemicellulosic fraction, inhibitors were also analyzed in the hydrolysate, because of their influence on fermentative processes. Researchers have reported that for concentrations above 2.0 g·L^−1^ of furfural, 2.0 g·L^−1^ of HMF, and 3.0 g·L^−1^ of acetic acid, the fermentative process is impaired when microorganisms of the genus *Candida* ssp. are used [17].

### 2.2. Lignocellulosic Composition

Utilizing hemicellulosic fraction to produce biofuels from sisal is a way of valorizing these carbohydrates making the process economically feasible, by utilizing a biomass that has high levels of fermentable sugars in its structure. Before performing the acid pretreatment, the sisal fiber was milled. Then, the lignocellulosic composition was determined. Table 3 also shows the percentage values of sisal fiber after the acid pretreatment, in the best conditions of the proposed experimental design.

The sisal fiber in natura has a high content of cellulose and hemicelluloses (31.81%). The low content of extractives and ashes, about 3%, suggests the presence of small amounts of organo-soluble compounds and minerals. The hemicellulose value is considered high since some studies using sisal have shown percentages between 10% and 20%, indicating high xylose content. The chemical composition may vary according to the origin of the material [27,28]. Sisal fiber presents a significant carbohydrate content (80%) and a small amount of lignin. This indicates fewer impediments to making the carbohydrates soluble, and thus, sisal fiber is shown to be a material for which acid pretreatment can be more effective and can solubilize a larger volume of sugar monomers.

The solid recovered after pretreatment was analyzed, and it was found that about one-third of the crude fiber was solubilized. According to the composition data after pretreatment (Table 3), about 19% of the hemicellulose of the solid sample was removed, and the hydrolyzed liquor (Table 4) in the best condition analyzed contained 80% pentoses, acetyl, and furfural groups (inhibitor derived from degradation of pentoses such as xylose)—that is, compounds that make up the hemicellulosic fraction of the sample. Much of the cellulose and lignin remained in the solid material (Table 3); only a small fraction of the cellulose was solubilized, which was probably a cellulose region with a lower crystallinity index that was more susceptible to acid cleavage [29]. These percentages indicate that the major components recovered by acid pretreatment were from the hemicellulosic fraction of the sample, as demonstrated in the study by Saleh et al. [30] for the recovery of sugars by treatment with dilute acid. The amount removed in some cases was 14% of the hemicellulose of the sample, but with temperatures above 172 °C. The present study used lower temperatures, maximum 120 °C, with satisfactory recovery results. Some researchers have shown higher biomass hemicellulose removal rates through increased acid concentration and operating temperature, but this increased the production of inhibitors for the fermentation process, and it is necessary to perform detoxification before fermentation. With the conditions used and the results obtained in this work, it was possible to carry out the fermentation process without the detoxification step of the hydrolyzed liquor, reducing the total costs of the process [14,17].

The post-treatment data indicate a satisfactory sugar recovery yield since the obtained hydrolysate contains 17 g of sugars per 100 g of sisal (Table 4). This percentage is considered satisfactory when compared with data in the literature for pretreatment with dilute acid, suggesting recovery percentages between 10 and 16 g of sugars per 100 g of biomass under higher temperature conditions than in the present study, in addition to a greater amount of furfural and HMF [14,30].

### 2.3. Infrared Characterization

**Figure 2:**
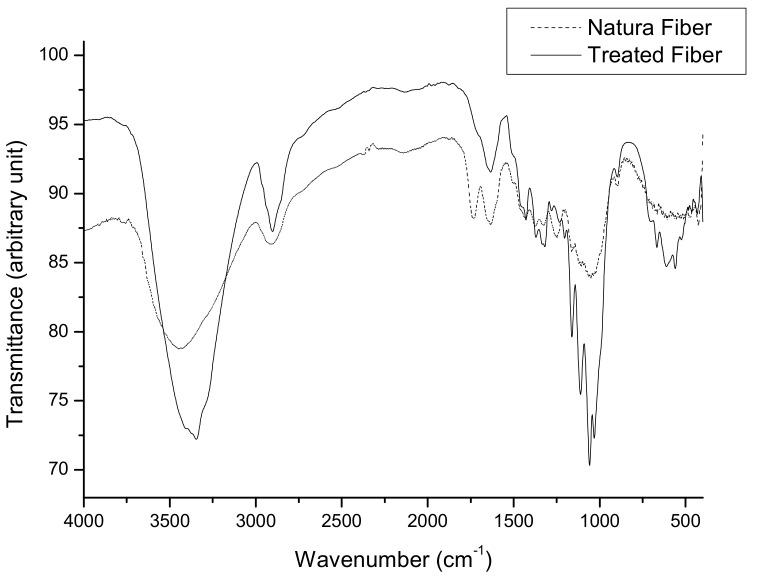
Infrared spectra of the sisal fiber.

The absorption spectra in the infrared region of the sisal samples in natura and after acid pretreatment are shown in Figure 2.

In the in natura samples, the bands located in the region of 1742–1730 cm^−1^ stand out. These may be attributed to the stretching of C=O bonds present in the ester bonds and/or in the carboxylic acids of the hemicellulose fraction [29,30]. The range between 1335 and 1380 cm^−1^ is formed by bands of vibrations of groups C–H and stretches O–H belonging to components of lignin and hemicellulose [29,31]. The composition of the hydrolyzed liquor (Table 4) shows the recovery of acetic acid and xylose monomers in greater quantity, indicating the cleavage of the branched chains of hemicellulose and their respective side groups. After the acid treatment was verified in the infrared spectrum of the solid material, the absence of the band at 1742 cm^−1^ suggested the removal of hemicellulose components [32] and the cleavage of acetate groups.

### 2.4. Characterization by Scanning Electron Microscopy

**Figure 3:**
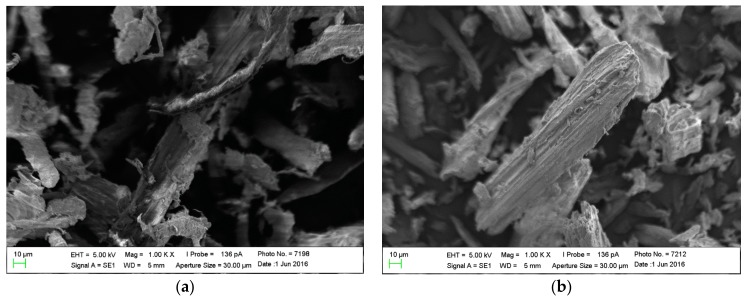
Micrographs of the sisal fiber: in natura (**a**) and treated (**b**). EHT: electronic acceleration; Mag: magnification; SE1: secondary electron detector; WD: working distance.

The micrographs of both the sisal fiber in natura and after acid pretreatment indicated the morphology of the fibers (Figure 3a,b).

In the in natura samples (Figure 3a), it is possible to observe individual bundles of fibers interlaced with the lignin-hemicellulose complex. In a very similar manner,during the study of sisal fiber as polymers’ reinforcement, it was found that samples of crude sisal fiber were composed of individual fibers, bonded together by cementing materials such as lignin, hemicellulose, wax, and oils [33].

After the pre-treatment (Figure 3b), the outer layer of the fibers was removed and the more complex structure, which existed before, was cleaved, making the cellulose fibrillar structure visible. According to Lacerda et al. [15], the gradual reduction in the sizes of the fibers is justified by the interaction of the acid in the lignocellulosic matrix. As a result, the fiber would be composed of smaller units, which makes it more susceptible to subsequent acid or enzymatic hydrolysis [34].

### 2.5. Characterization by X-ray Diffraction

**Figure 4:**
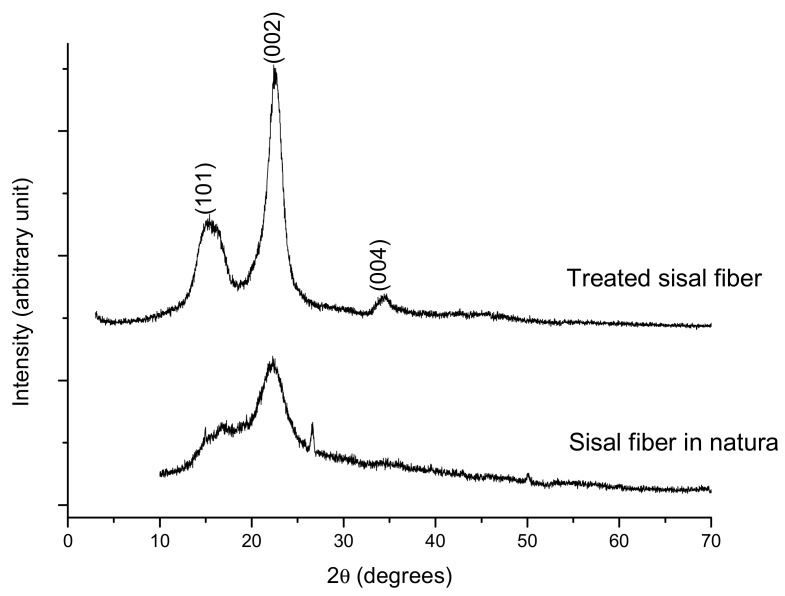
Diffractograms of the sisal fiber.

Sisal is considered a lignocellulosic composite, essentially consisting of the polymers cellulose, hemicellulose, and lignin. Only cellulose has a crystalline structure. The other constituents of the sample are of an amorphous nature [31]. The diffractograms (Figure 4) were analyzed to determine the crystallinity index of the treated and in natura samples.

As a result of the cellulose structures, the lignocellulosic materials had highly ordered regions and less-ordered regions [35]. In this sense, the acid pretreatment demonstrated the removal of amorphous components and thus increased crystallinity (78.50% treated sisal fiber), resulting in more intense peaks in the diffractograms. The in natura sample showed the lowest rate of crystallinity (44.89%) and increased noise (due to widening of the baseline), confirming the presence of amorphous regions. The observed increased crystallinity is probably a result, to a certain extent, of hemicellulose removal but could also be a possible contribution from the enhanced crystallization due to relaxation of the structure during the acid treatment, which is a phenomenon commonly observed for the acid treatment of lignocelluloses [36].

### 2.6. Fermentation

While planning for the best conditions for pretreatment, no production of acetic acid was anticipated. Once the best conditions were selected, the scale of production was expanded. A degradation of hemicellulose was observed, generating acetic acid in the fermentation medium (0.0586 g·g^−1^) [11]. The composition of the hydrolyzed liquor is described in Table 4. In addition to acetic acid, the liquor presented 0.0010 g·g^−1^ of HMF and 0.0083 g·g^−1^ of furfuralper gram of sisal fiber. Acetic acid is a by-product that is derived from the cleavage of acetyl groups present in the hemicellulose during the acid treatment. According to Rodrigues et al. [37], fermentation is stimulated by low concentrations of acetic acid, that is, those lower than 1.0 g·L^−1^. The concentration of acetic acid in the hydrolysate (Table 4) was higher than the amount that could stimulate fermentation. However, the production of xylitol and ethanol occurred without interference that can be attributed to acetic acid.

Since the microorganism used (*Candida tropicalis*) metabolizes xylose to produce xylitol and ethanol, it was verified that the liquor of the sisal fiber contains a significant quantity of this sugar (0.1229 g·g^−1^), in addition to 0.0462 g·g^−1^ of glucose per gram of sisal fiber.

**Figure 5:**
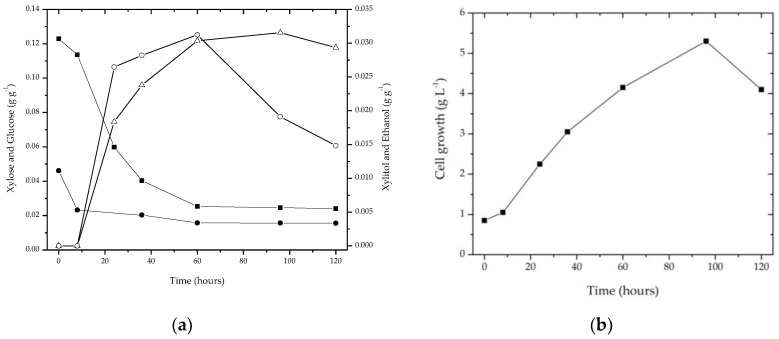
(**a**)Fermentation profile in the sisal fiber hydrolysate: (■) xylose, (∆) xylitol, (○) ethanol, (●) glucose; (**b**) Cell growth.

In the fermentation profile of the sisal fiber hemicellulose hydrolysate (Figure 5a), the consumption of xylose, and the production of xylitol and ethanol, were observed after cultivation in the fiber’s hydrolysate liquor for 120 h, using the yeast *Candida tropicalis* CCT 1516. As for the sisal fiber liquor, it was observed that during the first 8 h of fermentation, there was only consumption of sugars. At 24 h, when the concentration of xylose decreased to approximately 0.06 g·g^−1^, xylitol and ethanol were produced (0.018 g·g^−1^ and 0.026 g·g^−1^, respectively). The maximum simultaneous production of xylitol (0.0305 g·g^−1^) and ethanol (0.0312 g·g^−1^) was achieved in 60 h. After this, the concentration of ethanol decreased. By analyzing the cell growth (Figure 5b), it was observed that while the cells continue to grow, there was an increase in xylitol production. In a linear manner, as xylose was consumed, the production of xylitol increased. There was a small consumption of glucose during fermentation. After 96 h, xylitol decreased.

The conversion of xylose to ethanol can be stimulated in relation to the levels of other sugars present. According to Walther et al. [38], concentrations of monomeric sugars in the hydrolyzed liquor, used as a fermentation medium, may cause osmotic stress. This can inhibit the generation of the enzyme xylose reductase, or lead to the production of ethanol by the yeast in intolerable concentrations, in addition to affecting the kinetics of the enzymes present. This effect was observed in the fermentation profile of sisal hydrolysate, with simultaneous production of xylitol and ethanol. At the time of maximum yield, xylitol production was 0.32 g·g^−1^ per gram of xylose. Ethanol is a metabolite of the final conversion of xylose present in the medium, with a maximum concentration of 0.0312 g·g^−1^ of sisal fiber and a fermentation efficiency of approximately 47.8%, with a production of 0.27 g·g^−1^ ethanol per gram of consumed sugars. In the end, it was observed that 95% of the xylose present in the medium was consumed.

After 60 h of fermentation, the concentrations of xylitol and ethanol decreased (Figure 5a). According to Parajó et al. [39], the yield factors of products are dependent on the regulation of carbon flux through metabolic pathways. At the moment the sugars become scarce, the microorganisms use the alcohols as a carbon source, reducing the concentration in the medium.

The xylitol production from the sisal fiber hydrolysate presented satisfactory values when compared with the literature data. Misra et al. [40] obtained a yield of 0.50 g·g^−1^ xylitol, using *Candida tropicalis* as a microorganism in the hemicellulosic hydrolysate of corn ear, but the liquor underwent a detoxification process. The sisal fiber hydrolysate used in the present study did not undergo inhibitor removal treatment, thus avoiding additional costs and steps. Albuquerque et al. [17] state that using activated charcoal and thermal processes removes on average 46% of the acetic acid present. In this way, a significant amount of inhibitors will still exist in the medium, even after detoxification treatment. According to Yuan et al. [14], detoxification reduces the amount of sugars, consequently decreasing the efficiency of fermentation. Another problem associated with detoxification is the need for additional equipment and/or the use of chemical reagents, increasing capital investment for the production of xylitol and ethanol [40,41].

Linares et al. [42], using rapeseed straw hydrolysate, obtained simultaneous productivity of xylitol and ethanol similar to this work, with 0.42 g·g^−1^ and 0.12 g·g^−1^, respectively. However, hydrolysate was used after detoxification. Without going through the detoxification process, the sisal fiber of the present work obtained a higher productivity of ethanol and a similar production of xylitol. Other biomasses widely used in industry, such as sugarcane bagasse, have demonstrated similar productivity under the same experimental conditions, with the same genus of yeast used [43]. With this, it is possible to infer that sisal fiber is a potential biomass for producing ethanol and xylitol, creating value with the use of hemicellulosic liquor without detoxification and without the additional steps of alkaline pretreatment.

## 3. Materials and Methods

### 3.1. Materials

Sisal was obtained from farmers in the city of Nova Floresta-Paraiba. The sisal fiber was dried, milled, and then characterized before and after treatment, according to the Embrapa [44] methodology for determining the composition of lignocellulosic samples.

### 3.2. Pretreatment

The pretreatment [29,45] consisted ofanalyzing the influence of the variables—acid concentration (C) and temperature (T)—on the solubility of the xylose present in the sisal fiber. Dilute sulfuric acid was used. To this end, a 2^2^ experimental design was carried out, with three repetitions in the central point. The input variables were concentration of sulfuric acid (0.5, 1.5, and 2.5%) and temperature (100, 110, and 120 °C). The response was the xylose concentration, whose value was determined by the averaging of two tests. Significance was assessed by analysis of variance (ANOVA), with confidence levels higher than 95% (*p* < 0.05).

The pretreatment was conducted in a Maitec stainless steel reactor for 1h, in a sisal fiber/acidic solution ratio of 1:10. Wastes resulting from this acid treatment were analyzed, as were the samples in natura.

### 3.3. High Performance Liquid Chromatography

The concentrations of xylose, glucose, acetic acid, xylitol, ethanol, HMF, and furfural were measured by high-performance liquid chromatography (HPLC) in the Biochemical Engineering Laboratory/UFCG. This was done in a system equipped with a ProStar 210 pump (Varian, CA, USA), a manual injector with a 20 µL loop, a ProStar 356 refractive index detector (Varian) for the sugars and alcohols, a 284 nm UV/visibleforthe aldehydes, and a Hi-Plex H stainless-steel analytical column (0.30 m × 7.7 mm; Varian). Column temperature was 60 °C; mobile phase was H_2_SO_4_ 0.005 mol L^−1^, with a flow rate of 0.6 mL min^−1^; and analysis time was 1 h [7].

### 3.4. Fourier Transform Infrared Spectroscopy

The treated and untreated samples were analyzed by Fourier Transform Infrared Spectroscopy (FTIR) in a Prestige spectrophotometer (Shimadzu, Kyoto, Japan), using KBr in the range of 4000–400 cm^−1^ [31].

### 3.5. Scanning Electron Microscopy

The morphology and the change in physical structure of the sisal fiber, before and after the acid pretreatment, were evaluated by scanning electron microscopy (SEM). The samples were pulverized and metallized with gold. The analysis was carried out in a Leo 1430 Scanning Electron Microscope (Carl Zeiss, Oberkochen, Germany) in the voltage of 5 kV [33].

### 3.6. X-ray Diffraction

The X-ray diffraction (XRD) analyses were performed on a XRD 600 diffractometer (Shimadzu) with a CuKα radiation source, a voltage of 4 0 kV, a current of 30 mA, and a scan rate of 2° min^−1^ over a 2θ range of 10–80° (Fuel and Materials Laboratory/UFPB).

The cellulose’s crystallinity index [46] was determined through the equation:

(1) $$Ic=\left[\frac{I_{002}-I_{a}}{I_{002}}\right]\times 100$$

where *I_c_* is the intensity in a 2θ angle close to 22°, representing crystalline material; and *I_a_* is the intensity at 2θ angle close to 18°, representing amorphous material in the cellulosic fiber [7].

### 3.7. Preparation of the Inoculum

The yeast *Candida tropicalis* CCT 1516 was used in the experiments, and was donated by the Bioengineering Laboratory/UFPB from the Fundation Andre Tosello [47]. The pre-inoculum was made based on a mixture of 0.02 kg of rice bran in 1 L of water, which was autoclaved and centrifuged. Then, 0.03 kg of xylose, 0.002 kg of ammonium sulfate, and 0.0001 kg of calcium chloride were added to the supernatant, finalizing the volume at 1 L of mixture. In sequence, the solution was transferred to smaller containers, and yeast from the yeast-malt extract agar (YMA) culture was added to the medium. The Erlenmeyer flasks were kept under an agitation of 200 rpm and a temperature of 30 °C for 24 h [48].

The fermentation started when the yeast mass reached 3 g·L^−1^, or 10^7^ cells mL^−1^. At this time, the yeasts contained in the synthetic medium were centrifuged at 6000 rpm for 0.25 h, re-suspended in sterile distilled water, and added to the pre-hydrolyzed liquor, which had a pH of 5.5.

### 3.8. Fermentation

The fermentation was carried out in 0.5 L Erlenmeyer flasks containing 0.2 L of hydrolysate. Samples of the medium were collected for dry weight analysis, in order to verify the yeast behavior during the fermentation process, and to analyze the contents of sugar and the products formed during the fermentation. The fermentation times considered for study were 0, 8, 24, 36, 60, 96, and 120 h. The flasks were kept under agitation of 200 rpm and at a temperature of 30 °C. In order to analyze the behavior of the microorganism and to reduce the costs of the process, the fermentation was evaluated without the addition of nutrients. The nutrients were only added in the inoculum. Some research indicates that the addition of nutrients to the microorganism *Candida tropicalis* contributes to a small increase in productivity [14,41].

The yield in the conversion to xylitol/ethanol was calculated by the ratio between the product obtained and the xylose consumed. The other kinetic calculations were performed following the methodology described by Hickert et al. [41], Linareset al. [42], and Albuquerque et al. [17].

## 4. Conclusions

The sisal fiber presented a high content of carbohydrates and a small content of lignin. Acid pretreatment of sisal fiber can be more effective and can solubilize a larger amount of sugar monomers. The highest xylose contents were obtained at low temperature and acid concentration.

Without going through the process of detoxification, the sisal fiber of the present work obtained a higher productivity of ethanol and a similar production of xylitol when compared to other biomasses widely used in industry, such as sugarcane bagasse. In the sisal fiber hemicellulose hydrolysate, the maximum productions of xylitol (0.32 g·g^−1^ per gram of xylose) and of ethanol (0.27 g·g^−1^ per gram of consumed sugars) were achieved in 60 h. Thus, sisal fiber is presented as a potential biomass for the production of ethanol and xylitol, creating value with the use of hemicellulosic liquor without detoxification and without the additional steps of alkaline pretreatment.

## Figures and Tables

**Table 1:** Experimental design and amount of xylose extracted by acid pretreatment.

N°	Concentration (%)	Temperature (°C)	Xylose (g·g^−1^ of Sisal Fiber)
1	0.5 (−1)	100 (−1)	0.030
2	2.5 (+1)	100 (−1)	0.110
3	0.5 (−1)	120 (+1)	0.124
4	2.5 (+1)	120 (+1)	0.132
5	1.5 (0)	110 (0)	0.111
6	1.5 (0)	110 (0)	0.126
7	1.5 (0)	110 (0)	0.111

**Table 2:** Analysis of variance (ANOVA).

Source	DF	SS	MS	*F*-Test
Regression	3	0.6588	0.2196	1.04
Residual Error	3	0.0678	0.0226	
Lack-of-Fit	1	0.0519	0.0519	
PureError	2	0.0159	0.00798	
Total	6	0.7267		
% *R*^2^		90.66		
Regression Model	Xylose = 0.106 + 0.022 C + 0.029 T			

DF: degrees of freedom; SS: sum of squares; MS: mean of squares; C: concentration; T: temperature.

**Table 3:** Lignocellulosic composition of the sisal fiber.

Analysis	Natura Sisal Fiber/%	* Treated Sisal Fiber/%
Moisture	6.27	-
Ash	1.20	-
Extractives	1.75	-
Hemicellulose	31.81	23.70
Lignin	11.07	17.65
Alpha cellulose	48.20	38.87

* Composition after pretreatment at 120 °C and acid concentration of 2.5% (*v*/*v*).

**Table 4:** Composition of the sisal fiber hydrolysate.

Components	Concentration in the Liquor (g·g^−1^ of Sisal Fiber)
Glucose	0.0462
Xylose	0.1229
Acetic acid	0.0586
Hydroxymethylfurfural	0.0010
Furfural	0.0083
